# Supplementary material for: Barriers and facilitators to anti-retroviral therapy adherence among adolescents aged 10 to 19 years living with HIV in sub-Saharan Africa: A mixed-methods systematic review and meta-analysis
Source: PLoS One. 2023 May 18;18(5):e0276411. doi: 10.1371/journal.pone.0276411 (PMC10194875; doi:10.1371/journal.pone.0276411)
Supplement: S1 Table — (DOCX) [file pone.0276411.s001.docx]

**S1 Table. Search strategy for the systematic literature review**

| Search Number | Search words |
| --- | --- |
| 1 | Antiretroviral therapy OR ART AND Adherence OR Compliance |
| 2 | Barriers OR Challenges |
| 3 | ART outcome OR viral load suppression OR Loss to follow up |
| 4 | AND adolescents OR 10 to 19 years old |
| 5 | Angola OR Benin OR Botswana OR Burkina Faso OR Burundi OR Cabo Verde OR Cameroon OR Central African Republic OR Chad OR Comoros OR Congo, Democratic Republic OR Congo, Republic OR Cote D'ivoire OR Equatorial Guinea OR Eritrea OR Eswatini OR Swaziland OR Ethiopia OR Gabon OR Gambia OR Ghana OR Guinea OR Guinea-Bissau OR Kenya OR Lesotho OR Liberia OR Madagascar OR Malawi OR Mali OR Mauritania OR Mauritius OR Mozambique OR Namibia OR Niger OR Nigeria OR Rwanda OR Sao Tome and Principe OR Senegal OR Seychelles OR Sierra Leone OR Somalia OR South Africa OR South Sudan OR Sudan OR Tanzania OR Togo OR Uganda OR Zambia OR Zimbabwe OR Sub-Saharan African OR sub-Saharan Africa |
| 6 | 1 AND 2 AND 3 AND 4 AND 5 |
| 7 | 1 AND 4 AND 5 |
| 8 | 3 AND 4 AND 5 |
| 9 | 1 AND 2 AND 4 AND 5 |
