# Supplementary material for: Barriers and facilitators to anti-retroviral therapy adherence among adolescents aged 10 to 19 years living with HIV in sub-Saharan Africa: A mixed-methods systematic review and meta-analysis
Source: PLoS One. 2023 May 18;18(5):e0276411. doi: 10.1371/journal.pone.0276411 (PMC10194875; doi:10.1371/journal.pone.0276411)
Supplement: S2 Table — (DOCX) [file pone.0276411.s002.docx]

**S2 Table. Result of quality assessment of included studies using the Mixed Method Appraisal Tool (MMAT)**

|  | Qualitative | | | | | RCT | | | | | NRT | | | | | Descriptive Quantitative | | | | | Mixed | | | | |  |
| --- | --- | --- | --- | --- | --- | --- | --- | --- | --- | --- | --- | --- | --- | --- | --- | --- | --- | --- | --- | --- | --- | --- | --- | --- | --- | --- |
| Author (year), Country | 1.1 | 1.2 | 1.3 | 1.4 | 1.5 | 2.1 | 2.2 | 2.3 | 2.4 | 2.5 | 3.1 | 3.2 | 3.3 | 3.4 | 3.5 | 4.1 | 4.2 | 4.3 | 4.4 | 4.5 | 5.1 | 5.2 | 5.3 | 5.4 | 5.5 | Score (%) |
| Abiodun 2021  Nigeria |  |  |  |  |  | Y | Y | Y | Y | Y |  |  |  |  |  |  |  |  |  |  |  |  |  |  |  | 100 |
| Bermudez 2016  Uganda |  |  |  |  |  |  |  |  |  |  |  |  |  |  |  | Y | Y | N | Y | Y |  |  |  |  |  | 80 |
| Bermudez 2018  Uganda |  |  |  |  |  | N | Y | Y | N | Y |  |  |  |  |  |  |  |  |  |  |  |  |  |  |  | 60 |
| Bitwale 2021  Tanzania |  |  |  |  |  |  |  |  |  |  |  |  |  |  |  | Y | Y | Y | N | Y |  |  |  |  |  | 80 |
| Bongfen 2020  Camerron |  |  |  |  |  |  |  |  |  |  |  |  |  |  |  | Y | U | N | Y | Y |  |  |  |  |  | 60 |
| Bulali 2018  Tanzania |  |  |  |  |  |  |  |  |  |  |  |  |  |  |  | Y | Y | Y | Y | Y |  |  |  |  |  | 100 |
| Bygrave 2012  Zimbabwe |  |  |  |  |  |  |  |  |  |  |  |  |  |  |  | Y | Y | Y | N | Y |  |  |  |  |  | 80 |
| Haghighat 2021  South Africa |  |  |  |  |  |  |  |  |  |  |  |  |  |  |  | Y | U | Y | U | Y |  |  |  |  |  | 60 |
| Chaudhury 2018  Tanzania |  |  |  |  |  |  |  |  |  |  |  |  |  |  |  | Y | Y | Y | Y | Y |  |  |  |  |  | 100 |
| Crowley 2020  South Africa |  |  |  |  |  |  |  |  |  |  |  |  |  |  |  | Y | Y | Y | N | Y |  |  |  |  |  | 80 |
| Cluver 2020  South Africa |  |  |  |  |  |  |  |  |  |  |  |  |  |  |  | Y | Y | Y | Y | Y |  |  |  |  |  | 100 |
| Cluver 2018  South Africa |  |  |  |  |  |  |  |  |  |  |  |  |  |  |  | Y | Y | Y | Y | Y |  |  |  |  |  | 100 |
| Moyo 2020  Zimbabwe |  |  |  |  |  |  |  |  |  |  | Y | Y | N | N | Y |  |  |  |  |  |  |  |  |  |  | 60 |
| Wakooko 2020  Uganda |  |  |  |  |  |  |  |  |  |  |  |  |  |  |  | Y | Y | Y | Y | Y |  |  |  |  |  | 100 |
| Natukunda 2017  South Africa |  |  |  |  |  |  |  |  |  |  |  |  |  |  |  | Y | Y | Y | N | Y |  |  |  |  |  | 80 |
| Umar 2019  Malawi |  |  |  |  |  |  |  |  |  |  |  |  |  |  |  | Y | Y | Y | N | Y |  |  |  |  |  | 80 |
| Willies 2019  Zimbabwe |  |  |  |  |  |  |  |  |  |  |  |  |  |  |  | Y | Y | N | N | N |  |  |  |  |  | 40 |
| Nasuuna 2018  Uganda |  |  |  |  |  |  |  |  |  |  | Y | Y | Y | N | Y |  |  |  |  |  |  |  |  |  |  | 80 |
| Jobanputra 2015  Eswatini |  |  |  |  |  |  |  |  |  |  | Y | Y | N | Y | N |  |  |  |  |  |  |  |  |  |  | 60 |
| Gross 2015  Zimbabwe |  |  |  |  |  |  |  |  |  |  |  |  |  |  |  | Y | N | N | Y | Y |  |  |  |  |  | 60 |
| Sithole 2018  Zimbabwe |  |  |  |  |  |  |  |  |  |  |  |  |  |  |  | Y | Y | Y | Y | Y |  |  |  |  |  | 100 |
| Tanyi 2021  Kenya |  |  |  |  |  |  |  |  |  |  |  |  |  |  |  | Y | Y | N | Y | N |  |  |  |  |  | 60 |
| Desta 2020  Ethiopia |  |  |  |  |  |  |  |  |  |  |  |  |  |  |  | Y | N | Y | N | Y |  |  |  |  |  | 60 |
| Firdu 2017  Ethiopia |  |  |  |  |  |  |  |  |  |  |  |  |  |  |  | Y | Y | N | Y | Y |  |  |  |  |  | 80 |
| Kabogo 2018  Kenya |  |  |  |  |  |  |  |  |  |  | N | Y | Y | Y | Y |  |  |  |  |  |  |  |  |  |  | 80 |
| Bvochora 2019  Zimbabwe |  |  |  |  |  |  |  |  |  |  | Y | Y | N | N | N |  |  |  |  |  |  |  |  |  |  | 40 |
| Zhou 2021  South Africa |  |  |  |  |  |  |  |  |  |  |  |  |  |  |  | Y | Y | N | N | Y |  |  |  |  |  | 60 |
| Denison 2020  Zambia |  |  |  |  |  |  |  |  |  |  | Y | Y | N | N | N |  |  |  |  |  |  |  |  |  |  | 40 |
| Munyani 2020  Namibia |  |  |  |  |  |  |  |  |  |  | Y | Y | N | N | Y |  |  |  |  |  |  |  |  |  |  | 60 |
| Natukunda 2019  Uganda |  |  |  |  |  |  |  |  |  |  |  |  |  |  |  | N | Y | Y | N | Y |  |  |  |  |  | 60 |
| Ndiaye 2013  Botswana |  |  |  |  |  |  |  |  |  |  |  |  |  |  |  | Y | N | Y | N | Y |  |  |  |  |  | 60 |
| Chawana 2017  Zimbabwe |  |  |  |  |  | N | Y | Y | N | Y |  |  |  |  |  |  |  |  |  |  |  |  |  |  |  | 60 |
| Mburu 2019  Kenya |  |  |  |  |  |  |  |  |  |  | Y | Y | N | Y | N |  |  |  |  |  |  |  |  |  |  | 60 |
| Matyanga 2016  Zimbabwe |  |  |  |  |  |  |  |  |  |  |  |  |  |  |  | Y | Y | Y | N | Y |  |  |  |  |  | 80 |
| Wyk 2020  South Africa |  |  |  |  |  |  |  |  |  |  |  |  |  |  |  | Y | Y | Y | N | Y |  |  |  |  |  | 80 |
| Gitahi-Kamau 2020  Kenya |  |  |  |  |  |  |  |  |  |  |  |  |  |  |  | N | N | N | N | Y |  |  |  |  |  | 20 |
| Ochieng 2015  Kenya |  |  |  |  |  |  |  |  |  |  | N | Y | Y | Y | Y |  |  |  |  |  |  |  |  |  |  | 80 |
| Vogt 2016  Zimbabwe |  |  |  |  |  |  |  |  |  |  | Y | Y | N | Y | Y |  |  |  |  |  |  |  |  |  |  | 60 |
| Jereme 2019  Ethiopia |  |  |  |  |  |  |  |  |  |  | Y | Y | Y | Y | Y |  |  |  |  |  |  |  |  |  |  | 100 |
| Meloni 2020  Nigeria |  |  |  |  |  |  |  |  |  |  |  |  |  |  |  | Y | N | N | N | Y |  |  |  |  |  | 40 |
| Okonji 2021  South Africa |  |  |  |  |  |  |  |  |  |  |  |  |  |  |  | N | Y | Y | N | Y |  |  |  |  |  | 60 |
| Brathwaite 2021  Uganda |  |  |  |  |  |  |  |  |  |  |  |  |  |  |  | Y | Y | N | N | Y |  |  |  |  |  | 60 |
| Enane 2021  Kenya | Y | N | Y | Y | Y |  |  |  |  |  |  |  |  |  |  |  |  |  |  |  |  |  |  |  |  | 80 |
| Jimu 2021  Zimbabwe | Y | Y | Y | Y | Y |  |  |  |  |  |  |  |  |  |  |  |  |  |  |  |  |  |  |  |  | 100 |
| Khumoro 2021  Uganda | Y | Y | Y | Y | Y |  |  |  |  |  |  |  |  |  |  |  |  |  |  |  |  |  |  |  |  | 100 |
| Kunapareddy 2014  Kenya | Y | Y | N | N | Y |  |  |  |  |  |  |  |  |  |  |  |  |  |  |  |  |  |  |  |  | 60 |
| MacCarthy 2018 | Y | N | Y | Y | Y |  |  |  |  |  |  |  |  |  |  |  |  |  |  |  |  |  |  |  |  | 80 |
| MacCarthy 2020 |  |  |  |  |  |  |  |  |  |  |  |  |  |  |  |  |  |  |  |  |  |  |  |  |  |  |
| 100Madiba 201809  Botswana | Y | Y | Y | Y | Y |  |  |  |  |  |  |  |  |  |  |  |  |  |  |  |  |  |  |  |  | 100 |
| Mutwa 2012  Rwanda | N | Y | Y | Y | Y |  |  |  |  |  |  |  |  |  |  |  |  |  |  |  |  |  |  |  |  | 80 |
| Orth 2021  South Africa | N | N | Y | Y | Y |  |  |  |  |  |  |  |  |  |  |  |  |  |  |  |  |  |  |  |  | 60 |
| Rencken 2021  South Africa | Y | Y | N | Y | Y |  |  |  |  |  |  |  |  |  |  |  |  |  |  |  |  |  |  |  |  | 80 |
| Ritchwood 2020  South Africa | Y | N | N | N | Y |  |  |  |  |  |  |  |  |  |  |  |  |  |  |  |  |  |  |  |  | 40 |
| Roberts 2022  Zambia | Y | Y | Y | Y | Y |  |  |  |  |  |  |  |  |  |  |  |  |  |  |  |  |  |  |  |  | 100 |
| Burns 2020  Malawi | Y | Y | Y | Y | Y |  |  |  |  |  |  |  |  |  |  |  |  |  |  |  |  |  |  |  |  | 100 |
| Appiah 2019  Ghana | Y | Y | Y | Y | Y |  |  |  |  |  |  |  |  |  |  |  |  |  |  |  |  |  |  |  |  | 100 |
| Apondi 2021  Kenya | Y | Y | Y | Y | Y |  |  |  |  |  |  |  |  |  |  |  |  |  |  |  |  |  |  |  |  | 100 |
| Denison 2015  Zambia | Y | Y | Y | Y | Y |  |  |  |  |  |  |  |  |  |  |  |  |  |  |  |  |  |  |  |  | 100 |
| Ankrah 2016  Ghana | Y | Y | Y | Y | Y |  |  |  |  |  |  |  |  |  |  |  |  |  |  |  |  |  |  |  |  | 100 |
| vanWyk 2019  South Africa | Y | Y | Y | Y | Y |  |  |  |  |  |  |  |  |  |  |  |  |  |  |  |  |  |  |  |  | 100 |
| Chory 2021  Kenya | Y | Y | Y | Y | Y |  |  |  |  |  | Y | N | Y | N | Y |  |  |  |  |  | N | Y | Y | Y | Y | 80 |
| Cluver 2015  South Africa | Y | Y | N | Y | Y |  |  |  |  |  |  |  |  |  |  | Y | Y | Y | N | Y | Y | Y | Y | Y | Y | 100 |
| Dulli 2018  Nigeria | Y | Y | N | Y | Y |  |  |  |  |  | N | Y | Y | N | Y |  |  |  |  |  | N | Y | N | Y | N | 40 |
| Falkao 2021  Mozambique | Y | Y | Y | Y | Y |  |  |  |  |  |  |  |  |  |  | Y | Y | N | Y | N | Y | Y | Y | Y | Y | 100 |
| Mesic 2019  Zambia | Y | Y | Y | Y | Y |  |  |  |  |  |  |  |  |  |  | Y | Y | N | N | Y | Y | Y | N | Y | Y | 80 |
| Nabukeera-Barungi 2013  Uganda | Y | Y | Y | Y | Y |  |  |  |  |  |  |  |  |  |  | Y | Y | Y | Y | Y | Y | Y | Y | Y | Y | 100 |
| Stangly 2021  Zambia | Y | Y | Y | Y | Y |  |  |  |  |  | N | Y | N | N | Y |  |  |  |  |  | N | Y | Y | Y | N | 60 |
| Mavhu 2013  Zimbabwe | Y | Y | Y | N | Y |  |  |  |  |  | Y | Y | N | N | Y |  |  |  |  |  | Y | Y | N | Y | Y | 80 |
| Nyogea 2015  Tanzania | Y | Y | Y | Y | Y |  |  |  |  |  | Y | Y | Y | N | Y |  |  |  |  |  | Y | Y | Y | Y | N | 80 |
