# Supplementary figures and images for: Barriers and facilitators to anti-retroviral therapy adherence among adolescents aged 10 to 19 years living with HIV in sub-Saharan Africa: A mixed-methods systematic review and meta-analysis
Source: PLoS One. 2023 May 18;18(5):e0276411. doi: 10.1371/journal.pone.0276411 (PMC10194875; doi:10.1371/journal.pone.0276411)

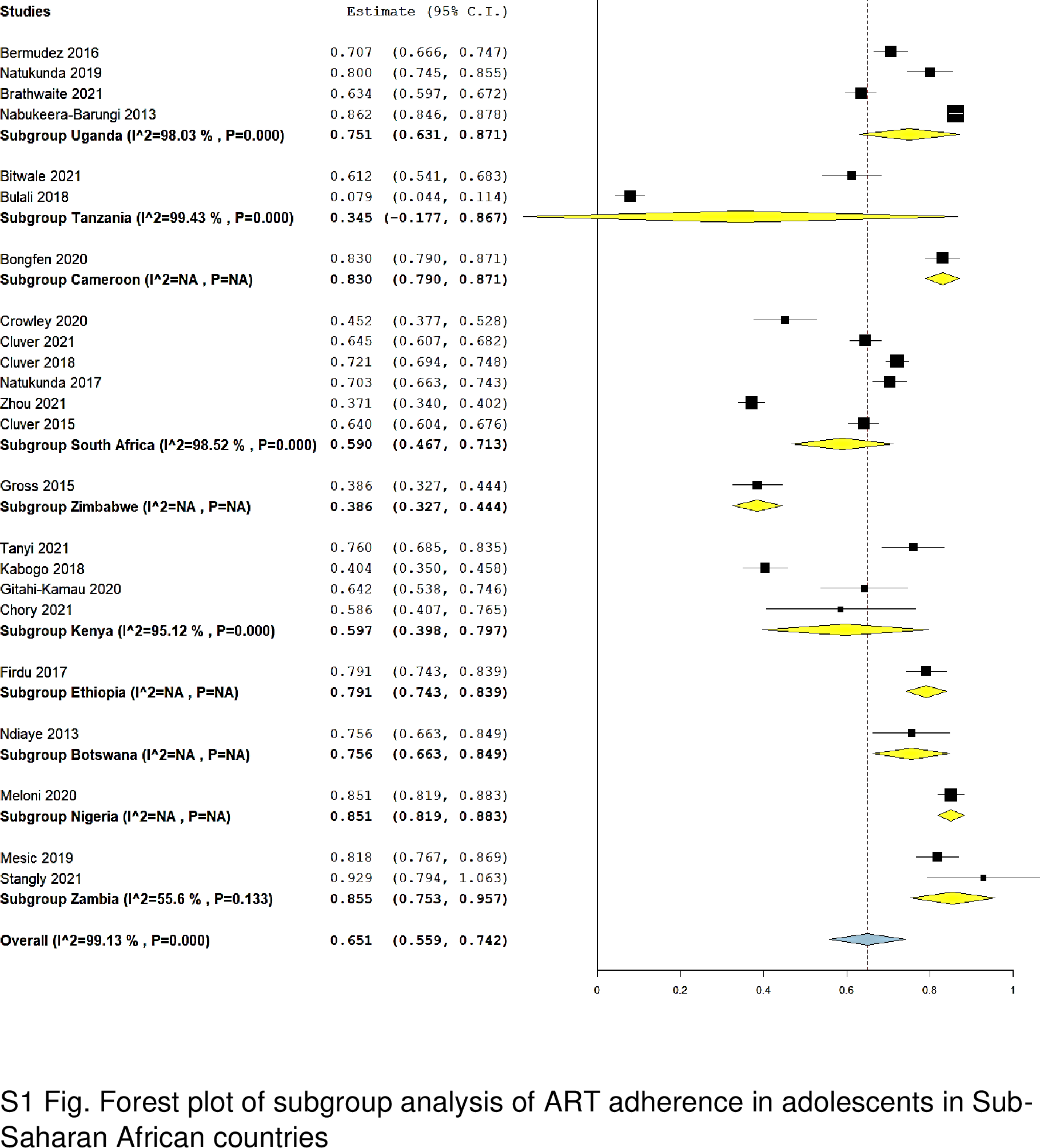

Supplement: S1 Fig — (TIF) [file pone.0276411.s004.tif]

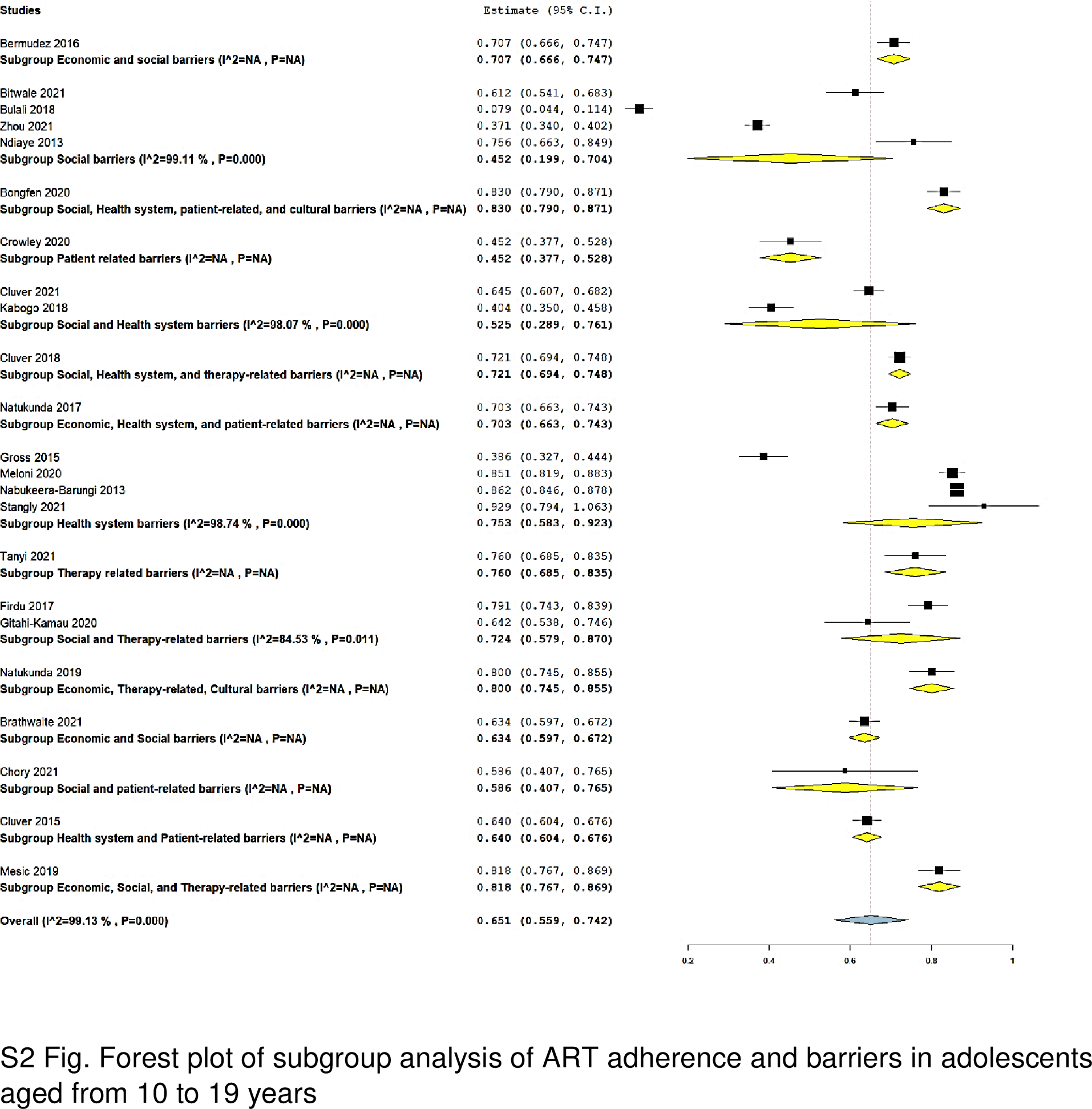

Supplement: S2 Fig — (TIF) [file pone.0276411.s005.tif]
