# Supplementary material for: Barriers and facilitators to anti-retroviral therapy adherence among adolescents aged 10 to 19 years living with HIV in sub-Saharan Africa: A mixed-methods systematic review and meta-analysis
Source: PLoS One. 2023 May 18;18(5):e0276411. doi: 10.1371/journal.pone.0276411 (PMC10194875; doi:10.1371/journal.pone.0276411)
Supplement: Attachment — Submitted filename: Review_ Charles Masulani Mwale.docx [file pone.0276411.s006.docx]

This is an important and timely study. The study is done according to the Preferred Reporting Items namely PRISMA statement, STROBE and ENTREQ recommendations.

The authors may consider the following:

1. Is there any reason why the authors did not consider viral suppression (another key outcome) in their search terms, while it is appearing in the methods section?
2. On the results included, only one study was from Malawi studies which is not the same as in the tables. There may be a need to crosscheck for the other countries as well.
3. The discussion could have been broadened to address all the significant results presented in the results section. Its short.
4. In the table for the studies included, from identification after removing the duplicates, to screening seems not to add up.
