# Supplementary material for: Barriers and facilitators to anti-retroviral therapy adherence among adolescents aged 10 to 19 years living with HIV in sub-Saharan Africa: A mixed-methods systematic review and meta-analysis
Source: PLoS One. 2023 May 18;18(5):e0276411. doi: 10.1371/journal.pone.0276411 (PMC10194875; doi:10.1371/journal.pone.0276411)
Supplement: Attachment — Submitted filename: Response to Reviewers.docx [file pone.0276411.s007.docx]

The Academic Editor

PLOS One

16th March 2023

Dear Editor

**RE: Rebuttal Letter**

I would like to extend our gratitude to your constructive comments received from both the Editor and Reviewer # 1 and 2.

I am hereby submitting a report below on the comments raised by both the editor and reviewers on manuscript titled “barriers and facilitators to anti-retroviral therapy adherence among adolescents aged 10 to 19 years living with HIV in sub-Saharan Africa: A mixed-methods systematic review and meta-analysis” during the review process.

We hope that we have addressed the comments satisfactory and that the manuscript will now be suitable for publication.

Best regards

Peter S Nyasulu PhD

Faculty of Medicine & Health Sciences

Stellenbosch University

Cape Town, South Africa

Phone: +27 21 938 9973

Email: [pnyasulu@sun.ac.za](mailto:pnyasulu@sun.ac.za)

Academic Editor:

1. **Please ensure that your manuscript meets PLOS ONE's style requirements, including those for file naming.**

The authors have edited the manuscript and have ensured that it is now in line with the journal’s requirements.

2. **"Please identify your study as "Systematic review and meta-analysis" in the title of your study.**

The authors have edited the title and the study is now identified as a systematic review and meta-analysis.

3. **"We note that Figure 2,  in your submission contain [map/satellite] images which may be copyrighted. All PLOS content is published under the Creative Commons Attribution License (CC BY 4.0), which means that the manuscript, images, and Supporting Information files will be freely available online, and any third party is permitted to access, download, copy, distribute, and use these materials in any way, even commercially, with proper attribution. For these reasons, we cannot publish previously copyrighted maps or satellite images created using proprietary data, such as Google software (Google Maps, Street View, and Earth). For more information, see our copyright guidelines:** [**http://journals.plos.org/plosone/s/licenses-and-copyright**](http://journals.plos.org/plosone/s/licenses-and-copyright)**.**

The map in our manuscript was created with Quantum Geographic information system (QGIS) software. We obtained African countries' shapefiles from a freely accessible database (https://www.naturalearthdata.com/downloads/). We geo-referenced the included studies in the shapefile based on their location as described in the table of included studies in our manuscript and joined the two files (shapefile and excel) to create the map. Based on the information provided, this map has not been published elsewhere and does not require permission

**Reviewer #1 Using the Assessment of Multiple Systematic Reviews (AMSTAR) Checklist, the authors have managed to include as much information on the checklist as possible (well done). While the authors have discussed how COVID-19 has affected HIV outcomes worldwide, it could be better if they add more literature on how this has affected the outcome of the included studies. Are there any differences in reported outcomes prior to COVID-19?**

While COVID-19 has been reported to have affected the HIV outcomes in general, our study could not be used to report on these effects or influences especially because some of the studies even though published post 2019/20, data was collected pre-COVID-19 and thus could be irrelevant in reporting the influence and impact of COVID-19 on adolescents ART adherence. However, authors have included more literature on the effects of COVID-19 on adolescents ART adherence.

Reviewer #2

1. **Is there any reason why the authors did not consider viral suppression (another key outcome) in their search terms, while it is appearing in the methods section?**

The authors have included the full search strategy as a supportive file (and viral load suppression and the other key ART outcomes have been included in the search strategy used for data collection.

2. **On the results included, only one study was from Malawi studies which is not the same as in the tables. There may be a need to crosscheck for the other countries as well.**

The results on studied included are disintegrated by the two different studies types (qualitative and quantitative). Therefore, there is one study under qualitative (line 301, Burns 2020) and one under quantitative (line 316, Umar 2019). In total, there are 2 studies from Malawi (one under qualitative studies and the other under quantitative studies). The authors also cross checked the other studies.

3. **The discussion could have been broadened to address all the significant results presented in the results section. Its short.**

Thank you, the authors have included more results in the discussion session including the meta-regression.

4. **In the table for the studies included, from identification after removing the duplicates, to screening seems not to add up.**

The authors have explained the observed discrepancies in lines 294-296 and lines 307-310. Kindly note that in the mixed methods studies, in some studies only one component was included in our analysis as the other component did not meet the eligibility criteria of our study for instance study population not integrated by age group especially in studies on adolescents and children or adolescents and adults.
